# The Fast and Reliable Detection of Multiple Narrowband FH Signals: A Practical Framework

**DOI:** 10.3390/s24154812

**Published:** 2024-07-24

**Authors:** Mutlu Aydin, Yaser Dalveren, Ali Kara, Mohammad Derawi

**Affiliations:** 1 Department of Electrical and Electronics Engineering, Gazi University, Ankara 06570, Turkey; mutlu.aydin@gazi.edu.tr (M.A.); akara@gazi.edu.tr (A.K.); 2 TUBITAK BILGEM ILTAREN, Sht Yzb Ilhan Tan Kislasi, Ankara 06800, Turkey; 3 Department of Electrical and Electronics Engineering, Izmir Bakircay University, Izmir 35665, Turkey; yaser.dalveren@bakircay.edu.tr; 4 Department of Electronic Systems, Norwegian University of Science and Technology, 2815 Gjovik, Norway

**Keywords:** frequency hopping, narrowband, parameter estimation, signal detection

## Abstract

Frequency hopping (FH) is a well-known technique that is commonly used in communication systems owing to its many advantages, including its strong anti-jamming capability. In this technique, basically, radio signals are transmitted by switching the carrier between different frequency channels. As a result, the FH signal is not stationary; hence, its spectrum is expected to change over time. Therefore, the task of detection and parameter estimation of FH signals is very challenging in practice. To address this challenge, the study presented in this article proposes a method that detects and estimates the parameters of multiple narrowband FH signals. In the proposed method, first, short-time Fourier transform (STFT) is utilized to analyze FH signals, and a practical binarization process based on thresholding is used to detect FH signals. Then, a new algorithm is proposed to ensure that the center frequencies of the detected signals are successfully separated. Next, another algorithm is proposed to estimate the parameters of the detected signals. After estimating the parameters for the entire spectrum, an approach is used to detect FH signals. Lastly, the hop-clustering process is applied to separate the hops into groups without time overlap. The simulation results show that the proposed method can be an efficient way for the fast and accurate parameter estimation and detection of multiple narrowband FH signals.

## 1. Introduction

The frequency-hopping spread spectrum (FHSS), also known as frequency hopping (FH), is a basic technique that is used in a typical wireless system to transmit radio signals by switching the carrier between different frequency channels. In general, the frequency band involves sub-frequencies. Between these sub-frequencies, the signal rapidly varies in a particular sequence, which is called “hops”. This reduces the interference of the signal. The FH is widely used in both civil and military communication systems due to its several advantages, including its strong anti-jamming capability and compatibility with other communication systems [1].

In practice, an electromagnetic interference (EMI) environment might be extremely complex because of the fixed frequency signals around the communication link as well as interference from nature. Since the FH signal is a non-stationary signal and its spectrum varies over time, the detection and parameter estimation of FH signals has become a very challenging issue. In turn, conventional signal processing methods may not be suitable to analyze the FH signals. In this case, time–frequency (TF) analysis, which is a two-dimensional joint method of time and frequency domains, could be an efficient method of estimating the parameters of FH signals [2,3,4,5,6].

In the literature, Wigner–Ville distribution (WVD) [7,8,9,10,11], wavelet transform [12,13], and short-time Fourier transform (STFT) [14,15,16,17] are the widely used TF analysis methods for FH signal parameter estimation. Among these methods, WVD has a higher TF resolution. However, it is computationally complex due to its cross-term interference. Wavelet transform has variable TF resolution, which is its main advantage over the other methods. Yet, as an important drawback, it is very sensitive to noise. On the other hand, STFT is not affected by cross-term interference. Thus, it can be considered to be the computationally simplest method in comparison to other methods. Due to its simplicity, STFT can be easily implemented. Therefore, STFT is the most often used method for analyzing FH signals.

On the other hand, FH is known as one of the important communication techniques in use by unmanned autonomous systems (UASs) due to its many advantages [18]. In particular, many unmanned aerial vehicles (UAVs) adopt FH signal-based transmission systems for secure communication. Hence, in recent years, most studies have been mainly focused on the estimation of UAV FH signals in an EMI environment to detect and classify UAVs, particularly in industrial, scientific, and medical (ISM) bands [19,20,21,22,23,24]. It is important to note that the frequency sequence of FH signals is typically fixed in real-world civil UAV applications. However, some communication systems conveying sensitive information may require some complex sequences, like pseudo-random hopping sequences. Therefore, the detection and accurate parameter estimation of FH signals remain challenging. Moreover, most of the studies in the literature that proposed FH signal parameter estimation methods have focused on the 2.4 GHz ISM operational band. However, FH signals carrying sensitive information might be narrowband in nature. Furthermore, latency is another important factor that needs to be taken into account to ensure the security of the protected area, platform, or system. Thus, it can be deduced that there are still many challenges for the fast and accurate parameter estimation of narrowband FH signals.

In this study, a fast and accurate approach is proposed to detect and estimate the parameters of multiple narrowband FH signals, which are commonly used in secure and sensitive message transmissions. In the proposed method, first, STFT was adopted to analyze the FH signals. After frequency transformation, a highly effective and practical binarization process based on thresholding was exploited to detect the FH signals. Next, a new algorithm was proposed to estimate the parameters of the detected signals. Following the bandwidth combining step, a new algorithm was proposed to refine the parameter estimation with the basic method. This new algorithm leverages the combined bandwidth and its center frequency information to achieve more accurate parameter estimation compared to the initial basic method. Once the parameters were estimated for the entire spectrum, hops were detected accordingly. Finally, in the last step, hop clustering was applied, which groups hops together while ensuring that they do not overlap in time. When adding a new hop to a group, the algorithm checks the bandwidths of both the new hop and the existing hops in the group. This ensures that only hops with non-overlapping time slots and compatible bandwidths are placed together. This approach effectively separates hops with the same bandwidth while preventing time overlaps. Simulations were conducted to examine the efficiency of the proposed method at different signal-to-noise ratios (SNRs). Two different frequency shift keying (FSK) modulation signals were used in the simulations. The results demonstrated that the proposed method could be an efficient means for the fast and accurate parameter estimation and detection of multiple narrowband FH signals.

The remainder of this paper is structured as follows. The next section presents a summary of related work and, in particular, the contribution of this study. In Section 3, the FH signal model is detailed. Then, the proposed method is described in Section 4. Section 5 describes the simulations performed to test and verify the proposed method. In Section 6, the results are discussed, and future research directions are addressed. Section 7 concludes this article.

## 2. Related Work and Contributions

In the literature, the parameter estimation and detection methods of FH signals can be divided into two main categories. On the one hand, methods are proposed for the detection and parameter estimation of a single FH signal [2,12,14,19,22,23,25,26,27,28,29,30,31]. On the other hand, some useful methods are proposed to be applied for multiple FH signals [4,20,24,32,33,34,35,36,37,38]. In this section, the relevant studies on the parameter estimation and detection methods of multiple FH signals are reviewed to discuss the main contributions of this study.

In [4], a blind parameter estimation algorithm based on space–time–frequency analysis (STFA) and matrix joint diagonalization (JDM) is presented. It was reported that the proposed algorithm could effectively estimate the parameters of multiple FH signals at higher SNR levels. In [20], a method based on an improved K-means clustering method was proposed to detect multiple FH drone signals. The experimental results demonstrated that the proposed method could be used to estimate the parameters of different FH drone control signals. In [24], a two-stage detection and classification method with goodness-of-fit (GoF), a deep residual neural network (DRNN), and a combined detection and classification with YOLO-lite methods were used to detect multiple FH signals. In [32], blind parameter estimation of multiple FH signals was proposed using a method that combined STFT and smoothed pseudo-Wigner–Ville distribution (SPWVD). It was shown that the hop period, timing, and center frequency parameters could be estimated for different networking modes. In [33], a method based on both compressed spectrum sensing and maximum likelihood (CSML) to estimate the frequency and hopping time parameters of multiple FH signals was proposed. It was verified that the proposed algorithm could achieve a fast and accurate estimation of multiple FH signal frequencies. In [34], a method based on a deep neural network (DNN) was proposed to detect multiple FH signals under low signal-to-noise ratio (SNR) conditions. In [35], a neural network-based signal sorting algorithm was presented to classify the FH description words of signals. According to the simulation results, it was shown that the proposed algorithm could provide better sorting accuracy in low SNR conditions. In [36], a deep learning-based parameter estimation method called the FH signal parameter extractor (FHExt) was proposed for the blind detection of multiple FH signals. According to the experimental results, it was verified that the FHExt could be accurate in fully blind scenarios. Moreover, it could be adaptable to semi-blind scenarios. In [37], a blind parameter estimation method based on few-shot learning for multiple FH signals was proposed. It was verified that the proposed method could achieve accurate parameter estimation even with a few annotations under various SNR conditions. Lastly, in [38], an improved object detector based on the YOLOv5 model using a ghost module to reduce computational cost was proposed to estimate both the TF parameters and 2D direction of arrival (DOA) of multiple FH signals in the uniform circular array.

A summary of the relevant works discussed above is shown in Table 1. As can be deduced from the table, only a few of the works used a test (signal frequency) band, which was around 2 GHz, to verify the efficiency of their proposed method. However, in this work, the 915 MHz frequency band is used for the performance evaluation of the proposed method for the first time in the literature. This suggests that the proposed method could be an alternative for the detection and parameter estimation of multiple FH signals in the very high frequency (VHF) and low ultra-high frequency (UHF) bands. Furthermore, the proposed method could detect multiple FH signals with different parameters, such as hop period, dwell time, and guard (frequency-switching) time. Moreover, a novel signal detection algorithm based on the use of the hop bandwidth parameter is proposed to avoid waiting for all TF values of the spectrogram.

## 3. Preliminaries

In this section, background information related to the FH signal model and STFT method is provided before introducing the proposed method. In this context, the characteristics of FH signals are represented first, followed by the description of the STFT method.

### 3.1. Frequency-Hopping Signal Model and Parameters

**Figure 1:**
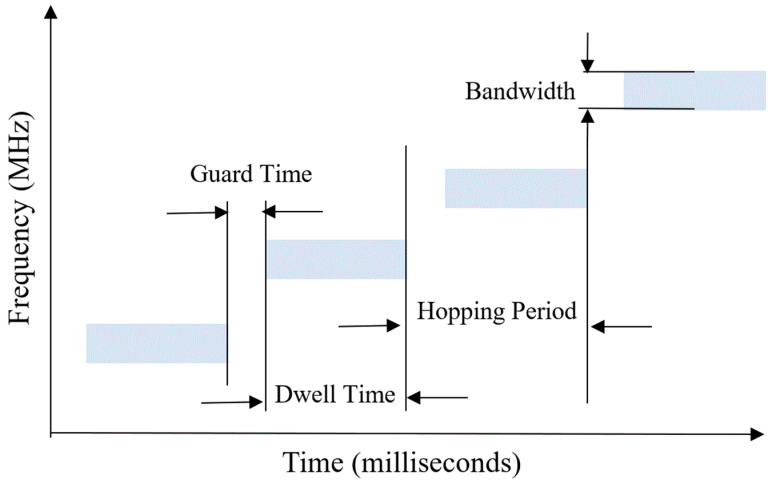
FH signal hop parameters.

The FH is known as a common type of spread spectrum (SS) signaling. This communication method uses a pre-established rule to convey the signals between the transmitter and receiver using a specific carrier frequency. Typical FH signal hop parameters are shown in Figure 1. As can be seen from the figure, one of the important parameters is the dwell time ($\tau_{dwell}$), which corresponds to the time between the start and stop times of the measured hop. The other one is the guard time ($\tau_{guard}$), which is the time between the end of the measured hop and the start of a new hop. Another is the hopping period ($T_{hopping}$), which is the sum of dwell time and guard time:

(1) $$T_{hopping}=\tau_{dwell}+\tau_{guard}$$

Moreover, using the hopping period parameter, the hopping rate parameter of a hop (HR) can also be calculated as follows:

(2) $$HR=\frac{1}{T_{hopping}}$$

Typically, FH signals can be stated as [19,39]

(3) $$x\left(t\right)=s\left(t\right)\sum_{m=0}^{M-1}\;e^{j2\pi f_{c_{m}}t_{m}+\theta_{m}}rect\left(\frac{t_{m}}{\tau_{{dwell}_{m}}}\right)$$

where $f_{c_{m}}$, $\theta_{m}$, $t_{m}$, and $\tau_{{dwell}_{m}}$ are the carrier frequency, carrier phase, hop duration length, and dwell time of the $m^{th}$ hop, respectively. Moreover, M is the total number of hops, and s(t) is the complex baseband equivalent of the information or message signal for t∈[0,T], where T is the total time of the FH signal.

The complex baseband equivalent of the received signal, on the other hand, can be expressed as

(4) $$r(t)=\sum_{n=0}^{N-1}\;y_{n}(t)+w(t)+I(t)$$

where $y_{n}(t)$ denotes an FH signal in the environment, w(t) represents complex additive white Gaussian noise (AWGN), where in-phase and quadrature components are independent and identically distributed (with zero-mean normal distribution), and $I\left(t\right)$ corresponds to interference signals within the receiver bandwidth.

### 3.2. Short-Time Fourier Transform

The STFT is an appropriate method to analyze FH signals. The STFT of a time-domain signal, z(t), can be mathematically expressed as [40]

(5) $$STFT\{z(t)\}=\int_{-\infty }^{\infty }\;[z(t)\omega (t-\tau )]e^{-j2\pi f\tau }d\tau$$

where ω(t) is a standard windowing function. In matrix form, here, the ith element of the STFT matrix, $S=\left[\begin{array}{cccc} s_{1}[f] & s_{2}[f] & \ldots & s_{K}[f] \end{array}\right]$, corresponding to a column vector, $s_{i}[f]$, can be determined by the discrete Fourier transform (DFT) of $r\left[n\right]\omega \left[n-iR\right]$ as follows [19]

(6) $$s_{i}[f]=\sum_{n=0}^{N-1}\;r[n]\omega [n-iR]e^{-j2\pi fn}$$

where R is the shifting length, r[n] and $\omega \left[n\right]$ are the sampled version of r(t) and ω(t), respectively.

The detection performance of the STFT is greatly dependent on the number of FH hops, dwell times, and time resolution. A minimum dwell time for a 100% probability of intercept (POI), $t_{100\%POI}$, can be calculated as [41]

(7) $$t_{100\%POI}=\left(2-P_{overlap}\right)\frac{N}{f_{s}},$$

where $P_{overlap\;}$ is the overlap ratio, $f_{s}$ is the sampling frequency, and N is the fast Fourier transform (FFT) window size.

## 4. Proposed Method

**Figure 2:**
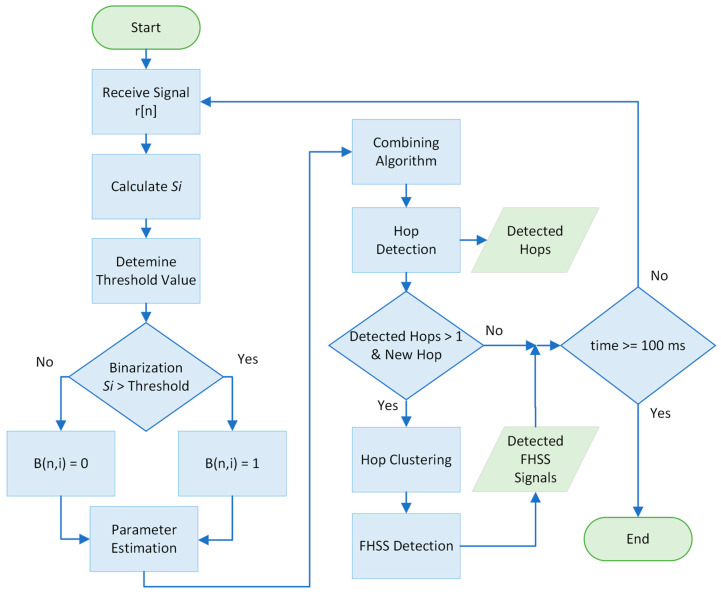
The flow chart of the proposed method.

A flowchart of the proposed method is depicted in Figure 2.

First, after receiving the signal (r(t)) STFT is applied to calculate $S_{i}$. It should be noted that FFT resolution ($f_{res}$) can be tuned according to the following ratio:

(8) $$f_{res}=\frac{f_{s}}{N}$$

In the proposed method, a Hamming window with a 4096 window size is used along with FFT [42]. In addition, a frequency resolution of 15 kHz is chosen in accordance with the Code of Federal Regulations (CFR).

After calculating the $S_{i}$, a binarization process similar to that used in [19] is implemented. In this process, the STFT matrix given in (5), S(n,i), is converted to a binarized matrix, $B\left(n,i\right)$. This is accomplished by calculating a threshold, φ, which is used for the identification of FH signals. The threshold calculation algorithm is given in Algorithm 1, where ${DG}_{dB}$ is the detection gap in dB units, and it can be selected according to the power of interference signals in the spectrum.
**Algorithm 1:** Threshold calculation algorithm**Input:** $S_{i}$**Output:** Threshold (φ)1. **if**
$S_{max}^{2}\geq \;median\left({S_{i}}^{2}\right)+{DG}_{dB}$ **then**2. $\varphi =\sqrt{max(median\left({S_{i}}^{2}\right)+{DG}_{dB},S_{max}^{2}-10dB)}$3. **else**4. $\varphi =\sqrt{median\left({S_{i}}^{2}\right)+{DG}_{dB}}$5. **end if**

Specifically, when the signal is detected, $B\left(n,i\right)$ becomes 1. Therefore, the binarized matrix can be mathematically expressed as

(9) $$B(n,i)=\left\{\begin{array}{ll} 1, & S(n,i)\geq \varphi \\ 0, & otherwise \end{array}\right..$$

The binarization process is followed by the estimation of signal parameters, such as center frequency and bandwidth. The algorithm that is used for this purpose is given in Algorithm 2. By means of this algorithm, signals from matrix B are detected, and the parameters are calculated accordingly. However, due to the channel impairments, there might be some distortions in matrix B. Thus, image processing-based methods are mostly utilized to recover the received signal [43]. These methods prompt the halting of the STFT process. Then, they detect slow FH signals with a long hopping period. Yet, fast-hopping signals can be identified quickly without having to wait extended periods of time. In the proposed algorithm, on the other hand, parameter corrections are employed at each stage of STFT. Additionally, the center frequency of each FH channel of FH signals is averaged. Thus, the algorithm refines its estimates as it progresses.
**Algorithm 2:** Parameter estimation algorithm in frequency domain**Input:** $B\left(n,i\right)$, n=0**Output:** center frequency ($f_{center}$), bandwidth (BW)1. **while**
*n* < N **do**2. **if** *B*(*n, i*) == 1 **then**3. $f_{start}$ = n $f_{res}-\frac{f_{s}}{2}$4. **while** *B*(*n + 1, i*) == 1 **do**5. $f_{stop}$ = $\left(n+1\right)\;f_{res}-\frac{f_{s}}{2}$6. *n* = *n* + 17. **end while**8. $f_{center}$ = ($f_{start}$ +$\;f_{stop}$ *)/2*9. BW = $f_{start}$ − $f_{stop}$ + $f_{res}$10. append $f_{center}$ and BW to a list11. **end if**12. *n* = *n* + 113. **end while**

In most real-world applications, the bandwidth of an FH signal includes a protection bandwidth, which is typically half of the bandwidth on either side of the center frequency. As all hops in an FHSS signal share the same bandwidth, a combining algorithm (Algorithm 3) verifies if the center frequencies of the detected signals are separated by a minimum distance. This minimum distance is determined by the sum of the bandwidth of the signals and the proportional protection bandwidth. The proportion of protection bandwidth ($P_{PBW})$ can be selected by the user ($0<P_{PBW}<1$). In this algorithm, the center frequencies are combined, providing that the separation is less than the sum of signal bandwidth and protection bandwidth. As a result, this paves the way for reducing the false alarm rate.

Once the channel center frequencies and bandwidths are estimated for the entire spectrum, hops are detected accordingly, and they are retained for further processing. If a hop cannot be detected, the process is continued for the next stage of STFT. The channel center frequencies and bandwidths are calculated as in the previous stages. However, the key difference lies in comparing the center frequencies of the estimated channels between the iterations. If a newly estimated center frequency falls within the range of a previously detected channel, the center frequencies of both estimates are averaged. The range of the detected channel is proportional to the estimated bandwidth. The bandwidth estimation of an FHSS signal can be distorted at the start and stop times of hops due to discontinuities in the signal at the FFT stage. The discontinuities cause the signal to spread across a wider range of frequencies in the spectrum. To eliminate such distortions, a specific algorithm is developed. The algorithm provides a selection of 97.5% of the lowest and highest estimated frequencies ($f_{stop},\;f_{start})$, and takes the maximum and the minimum, respectively. The updated bandwidth is then calculated as the difference between these two estimated values. In this way, bandwidth expansion distortions could be eliminated. In fact, this method helps to provide a more precise estimation of channel center frequencies as well as the signal bandwidths.

Furthermore, if no new information is added to a channel that was active at the previous stage, the hop-stop time of that channel is still stored. Hence, the start and stop time parameters of all hops can be estimated to be used in the hop-clustering stage.
**Algorithm 3:** Combining algorithm**Input:** center frequency list (*cfl*), bandwidth list (*bl*), *k* = 0**Output:** center frequency list (*cfl*), bandwidth list (*bl*)1.  **while**
*k* < length(*cfl*) – 1 **do**2.  *found* = **False**3.   **for**
*l* from *k*+1 to length(*cfl*) – 1 **do**4.     **if**
$\left|cfl\left(k\right)-cfl(l)\right|$ < max(*bl*(*k*), *bl*(*l*))×${(1+P}_{PBW})$ **then**5.      **if** $cfl\left(k\right)>cfl\left(l\right)$ **then**6.       $f_{start}$ = $cfl\left(k\right)+\frac{bl\left(k\right)}{2}$7.       $f_{stop}$ = $cfl\left(l\right)-\frac{bl\left(l\right)}{2}$8.     **else**9.       $f_{start}$ = $cfl\left(l\right)+\frac{bl\left(l\right)}{2}$10.       $f_{stop}$ = $cfl\left(k\right)-\frac{bl\left(k\right)}{2}$11.     **end if**12.     $f_{center}=\frac{\left(f_{start}+f_{stop}\;\right)}{2}$13.     $BW=\left(f_{start}-f_{stop}\right)$14.     delete $cfl\left(k\right)$, $cfl\left(l\right)$, $bl\left(k\right),$ and $bl\left(l\right)$15.     append $f_{center}$ to *cfl*16.     append BW to *bl*17.     *found* = **True**18.     **break**19.    **end if**20.    **if not** *found* **then**21.      k=k+122.    **end if**23.  **end while**

The detection of hops is followed by a hop-clustering process. In general, hops can be separated into groups based on whether they overlap in time or not. The main goal would be to separate hops without time overlapping [20]. After separating hops according to the time ranges, the bandwidths of hops are also inspected to detect hops with the same bandwidth. Yet, due to the estimation errors, a tolerance value needs to be determined. In this process, the parameter $f_{res}$ is selected as a tolerance value for bandwidth estimations to decide whether hops have the same bandwidth or not. Therefore, a time overlap-based clustering algorithm used in [20] is adapted to the proposed method (Algorithm 4).

In general, the dwell time of hops could be misleading. Frequency-hopping systems considered in this study switch their center frequencies in a pseudo-random manner. When guard time (frequency-switching time) is negligible, and consecutive repetitions of center frequencies are included, the dwell time at each frequency, which is a multiple of actual dwell time in this case, can be estimated simply as dwell time. Thus, dwell time cannot be used as a reliable discriminative parameter. Nevertheless, some other parameters, such as DOA, symbol rate, symbol sequence, energy, guard time, and modulation, could be used for hop clustering. The clustering process of the proposed method characterizes an FH signal by identifying its hop parameters. These parameters include center frequency, bandwidth, start and stop time, dwell time, guard time, and hop rate.
**Algorithm 4:** Hop-clustering algorithm**Input:** hop list**Output:** FH list1.  *sorted_hl =* sort hop list by *start time*2.  *count* = 0% used for *FH_ID*3.  **for** ${hop}_{sorted\_hl}$ **in** *sorted_hl* **do**4.   *placed* = **False**5.   **for**
${FH}_{i}$ **in** FH list **do**6.    *overlap =*
**False**7.    **for**
${hop}_{{FH}_{i}}$**in** ${FH}_{i}$ **do**8.      **if**
${start\;time}_{{hop}_{sorted\_hl}}\leq$ ${stop\;time}_{{hop}_{{FH}_{i}}}$ **then**9.        *overlap =*
**True**10.        **break**11.      **end if**12.    **end for**13.    **if not**
*overlap*
**then**14.      % for separating hops with different bandwidth15.      **if** $\left|{BW}_{{hop}_{sorted\_hl}}-{BW}_{{hop}_{0}of{FH}_{i}}\right|<f_{res}$ **then**16.      % ${BW}_{{hop}_{0}of{FH}_{i}}$ is the bandwidth of the first hop of the ${FH}_{i}$17.        append ${hop}_{sorted\_hl}$ to ${FH}_{i}$18.        *FH_ID* = index(i) of ${FH}_{i}$19.        *placed* = **True**20.        **break**21.      **end if**22.    **end if**23.   **end for**24.   **if not** *placed* **then**25.     append [${hop}_{sorted\_hl}$] to FH list26.     *FH_ID* = *count*27.     *count = count + 1*28.   **end if**29.   add *FH_ID* to ${hop}_{sorted\_hl}$ parameters30. **end for**


The final stage of the proposed method is the FH detection stage. The hops that emerge at a stage and then disappear at the next stage are eliminated to reduce the false alarm rate. Similarly, FH lists with only one hop are ignored to eliminate the false detection of continuous signals as well as interference signals. If a specific FH signal (threat) is intended to be detected, the range of the parameters of the signal can be provided to the FH detection algorithm within the tolerance values. The detection algorithm then returns an alarm for the identification of the threat when the threat appears in the reception system. If no pre-defined FH signal is given, the algorithm will return an alarm only for the identified FHSS signals at the clustering stage.

It is also worth noting that an STFT is computed within a very short time from the received data. Thus, the successful detection of slow hopping signals is considered in particular. However, increasing the STFT size may significantly increase the computational cost. For the sake of low computational cost, STFT may use no overlap (0%), sacrificing the time resolution. No overlapping (0%) gives rise to a time resolution ($T_{res}$) corresponding to the acquisition time of an FFT, which can be expressed as

(10) $$T_{res}=\frac{N}{f_{s}}.$$

## 5. Simulations

In this section, simulations are presented to demonstrate the effectiveness of the proposed method. First, the simulation parameters and environment are described. Then, the simulation results are discussed.

### 5.1. The Simulation Parameters and Environment

The simulation parameters were selected based on real-world implementations. This suggests the common practice of using low-cost software-defined radio (SDR)-based solutions for FH signal detection. Here, we explore the practical aspects of generating and detecting FH signals using commercial off-the-shelf (COTS) SDRs. This involves identifying the capabilities and limitations of such hardware platforms. However, the proposed framework is designed with extendibility in mind so that it allows adaptation to other hardware platforms and operational conditions. The following parameters were chosen by considering the capabilities of COTS SDRs in the market. First, the sampling frequency was selected to be 61.44 MS/s to align with the sampling rate of COTS SDRs as well as the targeted operational requirements of FH signals. Operational frequency could be selected from 70 MHz to 6 GHz in such SDRs. Hence, the operational frequency was chosen in this range with a 56 MHz instantaneous bandwidth. Thus, using (10), $T_{res}$ could be determined as around 66.7 µs. Furthermore, using (7), a minimum signal duration of 100% POI could also be determined as around 133.4 µs with a 0% overlap ratio. This would yield to detect FH signals with theoretical limits of up to 7500 hops/s. It is important to note that these can be provided for the threats at VHF and lower UHF bands, such as the 915 MHz ISM band. Before performing the simulations, ${DG}_{dB}=14$ dB was taken in Algorithm 1. Moreover, the $P_{PBW}$ was determined as 65% of the estimated bandwidth to be used in the combining algorithm (Algorithm 3). Again, this parametric configuration still offers flexibility that may allow for straightforward adaptation to various operational scenarios.

In the simulations, two FH signals were generated using the GNU Radio (v3.10) library, which is an open-source toolkit for signal processing utilized in the development and deployment of SDRs [21]. In this study, four-frequency shift keying (4-FSK) and eight-frequency shift keying modulation (8-FSK) schemes were used. The signal parameters, including center frequency ($f_{center}$), bandwidth (BW), dwell time ($\tau_{dwell}$), guard time ($\tau_{guard}$), and hopping rate (HR) are listed in Table 2.

It is important to note that the GNU Radio library was used to generate FSK signals in conjunction with Python’s NumPy library for signal modeling. Specifically, FSK signals were generated by GNU Radio, which was then used within Python to create FH signals. After generating FH signals, AWGN with various power levels was added to simulate different SNR values. 

**Figure 3:**
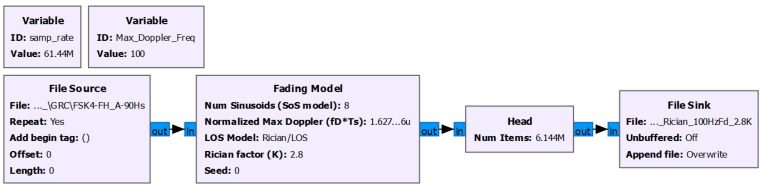
Rician fading model using GNU Radio companion.

Furthermore, it is important to acknowledge that this study focuses on air-to-land transmissions, where the channel can often be approximated as frequency-flat while experiencing Rician fading. Therefore, similar to the approach in the previous works of the authors [24], the performance of the proposed method was examined under Rician fading conditions. To consider Rician fading conditions in the simulations, GNU Radio companion was used. The corresponding flowgraph is shown in Figure 3. In the simulations, the Rician factor (K) and Doppler frequency ($f_{D}$) were selected as 2.8 and 100 Hz, respectively.

### 5.2. Results and Discussion

Simulations were performed to verify the effectiveness of the proposed method at different SNR levels. The SNR values were selected according to the selected FFT window size (N), as it is proportional to the FFT processing gain that reduces the noise floor [42]. Therefore, the SNR levels are considered to be −8 dB, 6 dB, and 24 dB in the simulations.

**Figure 4:**
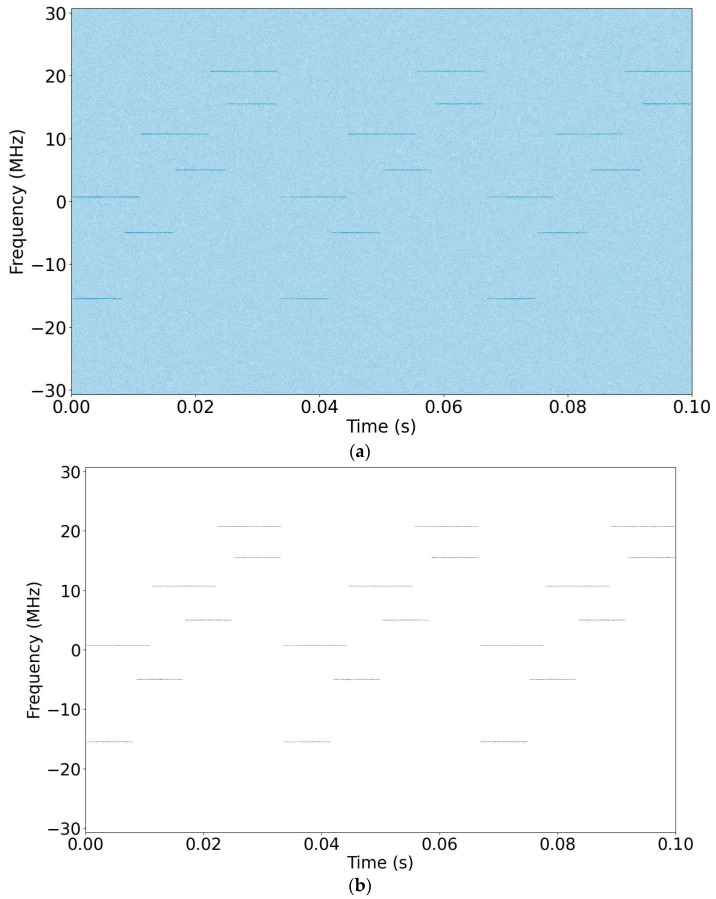
(**a**) A spectrogram of the complex baseband signal (SNR = −8 dB); (**b**) A binarized version of the spectrogram.

As an example, simulation results achieved at −8 dB SNR level for the initial stages of the proposed method are shown in Figure 4. Figure 4a shows the spectrogram of the complex baseband signal with Rician channel (K = 2.8 and $f_{D}$ = 100 Hz at −8 dB SNR) using an STFT with 0% overlap and a 4096-point Hamming window, while Figure 4b shows its binarized version.

**Figure 5:**
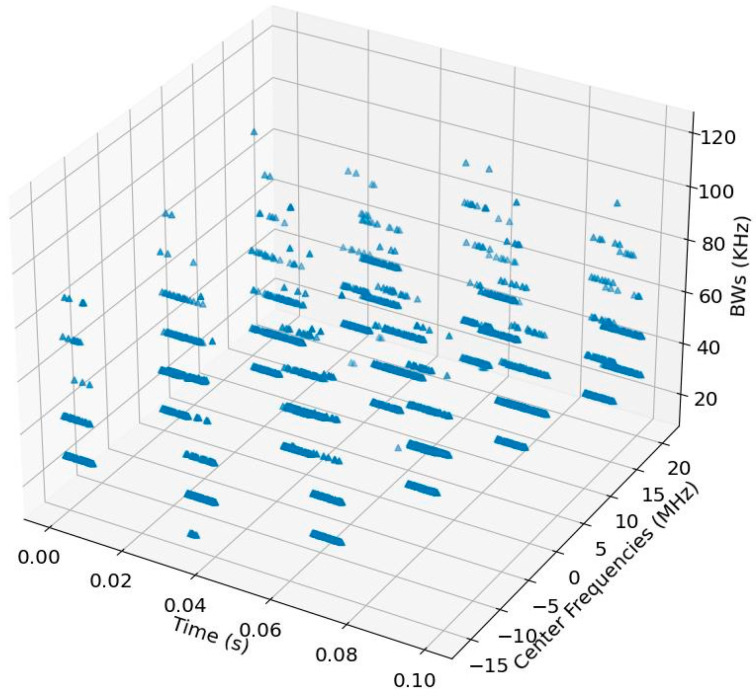
A 3-D scatter plot of all the estimated channel frequencies and corresponding bandwidths after the combining stage (SNR = −8 dB).

Overall, Figure 5 shows a 3-D scatter plot of all the estimated channel frequencies and corresponding bandwidths after the combining stage.

**Figure 6:**
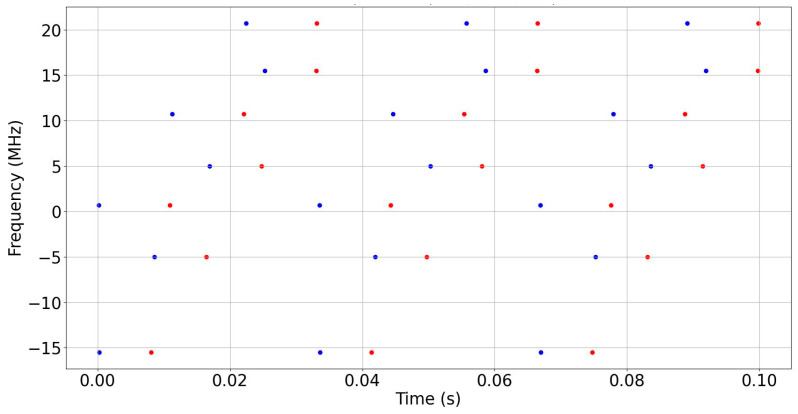
The start and stop times of the detected hops signal (SNR = −8 dB).

Moreover, the results achieved after the hop detection stage at −8 dB SNR are shown in Figure 6, where the blue circles denote the hop starts while the red circles denote the hop stops.

**Figure 7:**
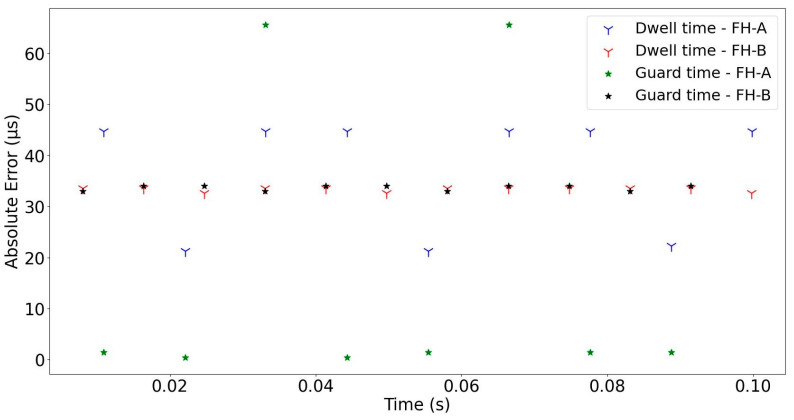
The absolute errors of the dwell time and guard time of the detected FH signals (SNR = −8 dB).

**Figure 8:**
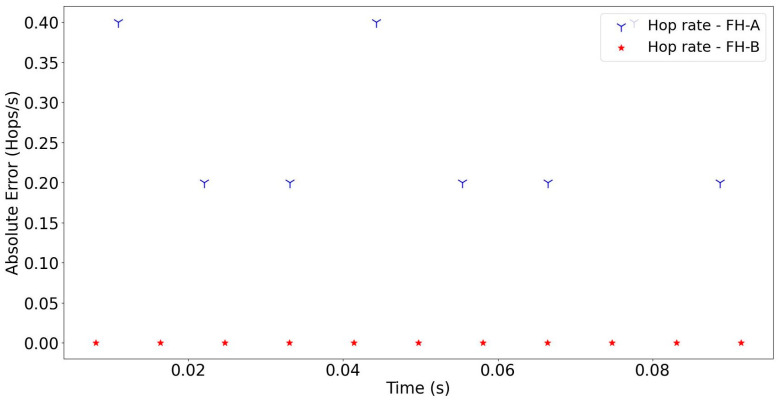
The absolute errors of the hopping rate of the detected FH signals (SNR = −8 dB).

Based on the results achieved from the experiments, the performance of the proposed method was examined in two steps. In the first step, the absolute error of the estimated signal parameters, such as dwell time, guard time, and hopping rate, was calculated to evaluate the performance of the proposed method. The absolute errors of the signal parameters estimated at −8 dB are shown in Figure 7 and Figure 8. Overall, it should be noted that the proposed method efficiently estimated all the parameters of both FH-A and FH-B signals. In particular, it performed well in separating the bandwidths even when they were set very close to each other. The proposed method offers a significant advantage in clustering overlapped FH signals in the time domain. This is due to the fact that bandwidth is a critical clustering parameter in multi-signal environments.

**Figure 9:**
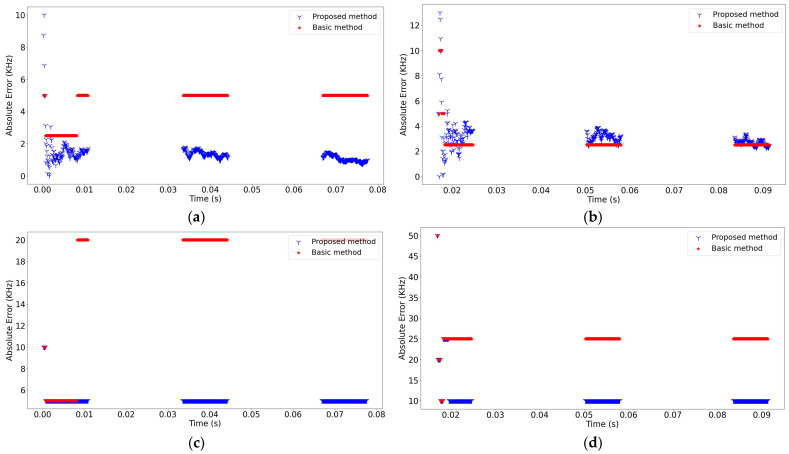
The absolute error comparison of the methods signals (SNR = −8 dB): (**a**) The absolute error of $f_{center}$ for the 0.7 MHz FH-A signal; (**b**) The absolute error of $f_{center}$ for the 5 MHz FH-B signal; (**c**) The absolute error of BW for the 0.7 MHz FH-A signal; (**d**) The absolute error of BW for the 5 MHz FH-B signal.

**Figure 10:**
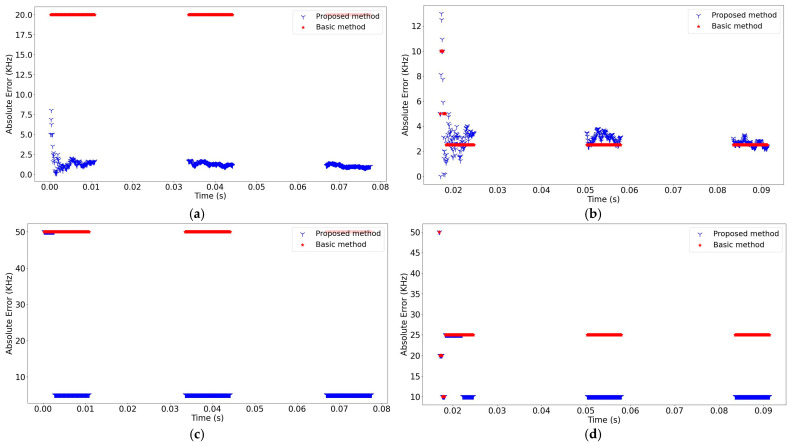
The absolute error comparison of the methods (SNR = 6 dB): (**a**) The absolute error of $f_{center}$ for the 0.7 MHz FH-A signal; (**b**) The absolute error of $f_{center}$ for the 5 MHz FH-B signal; (**c**) The absolute error of BW for the 0.7 MHz FH-A signal; (**d**) The absolute error of BW for the 5 MHz FH-B signal.

**Figure 11:**
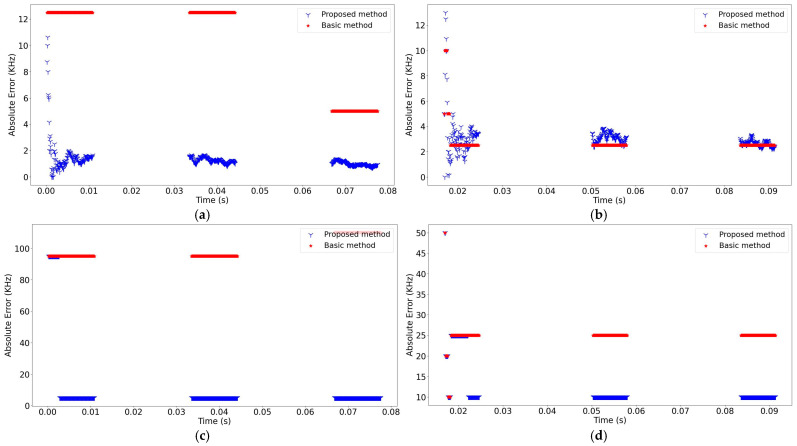
The absolute error comparison of the methods (SNR = 24 dB): (**a**) The absolute error of $f_{center}$ for the 0.7 MHz FH-A signal; (**b**) The absolute error of $f_{center}$ for the 5 MHz FH-B signal; (**c**) The absolute error of BW for the 0.7 MHz FH-A signal; (**d**) The absolute error of BW for the 5 MHz FH-B signal.

In addition to bandwidth, center frequency ($f_{center}$) is another critical parameter to be estimated in multi-signal environments. Therefore, the second step aimed at comparatively assessing the performance of the proposed method in estimating these parameters. To this end, the approach used in the proposed method, where the averaging was used for estimating $f_{center}$ and the eliminating (thresholding) was applied for BW estimation and was replaced with a traditional (basic) method that has been used in the literature. In the basic method, BW is calculated as the maximum value of the difference between the $f_{start}$ and $f_{stop}$ values, while $f_{center}$ is calculated by taking the average of the $f_{start}$ and $f_{stop}$ values that provide the maximum difference [20,32]. Then, the absolute error was used to evaluate the performance of the proposed method. For the 0.7 MHz FH-A signal and the 5 MHz FH-B signal, the calculated absolute errors of $f_{center}$ and BW at −8 dB, 6 dB, and 24 dB SNR levels are presented in Figure 9, Figure 10, and Figure 11, respectively. It is clear that the proposed method significantly increased the accuracy of parameter estimations.

**Figure 12:**
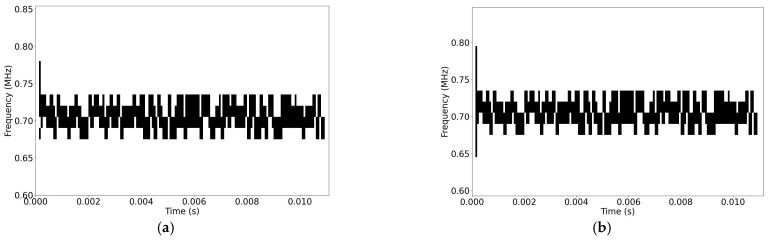
A comparison of the first hop of the binarized FH-A signal at (**a**) SNR = 6 dB; (**b**) SNR = 24 dB.

According to the results, it can be concluded that the absolute errors increase with increasing SNR. This can be linked with the thresholding technique, where the minimum detection threshold level is estimated to determine a threshold level. Additionally, a threshold for high SNR signals is calculated as 10 dB lower than the maximum power of the FH signals. The thresholding algorithm selects the greater of these two values. Therefore, if the maximum power is not 10 dB greater than the minimum detection threshold, the range of the selected threshold relative to the maximum power could be reduced. This leads to a reduction in the estimated bandwidth. The described bandwidth widening at the start and the stop of hops, due to the discontinuity at windows affected by the threshold level, might be mitigated at low SNR. In such cases, the reduction in bandwidth estimation due to a low SNR cancels out the increase caused by the widening effect. As an example, a comparison of the first hop of the binarized FH-A signal at 6 dB and 24 dB SNR is shown in Figure 12.

This initial research focused on air-to-land transmissions, where the channel could often be approximated as frequency-flat while experiencing Rician fading. In this context, for testing the proposed method under different Rician channels, varying K factors are implemented in the fading model. The results are shown in Table 3 in terms of average absolute errors (∆), which is quite consistent with the previous research [24].

To sum up, by significantly reducing waiting time during the detection, the proposed thresholding-based method offers a substantial advantage over traditional STFT methods for narrowband FH signal detection. In traditional STFT methods, detection algorithms need to wait for all spectrogram samples to be acquired and converted to the frequency domain with FFT. In the proposed method, this duration is reduced to only two hopping periods, which significantly reduces time consumption. At the end of the detection process of two narrowband FH signals, the proposed method reduces the waiting time to 22.2% (FH-A) and 16.7% (FA-B) of the sample acquisition and FFT processing times required by the traditional method.

## 6. Discussion and Future Work

The results achieved from the simulations verified that the proposed method in-creases the accuracy of the parameter estimations as well as the detection of multiple narrowband FH signals at various SNR levels. The proposed algorithm enables fast FH signal detection by strategically processing only the relevant portions of the STFT spectrogram. This efficient structure makes the proposed method suitable for real-time threat identification systems demanding rapid detection of fast FH signals.

There might still be some concerns regarding the performance of the proposed system in real-world scenarios. Power variations in the received FH signals, for example, may pose a difficulty to the thresholding process. However, real-world testing can easily refine the method for robust performance. Moreover, the proposed method could be further extended to estimate modulation type, symbol sequence, or FSK shift rate. This may improve the clustering of FH signals, in particular, bandwidth-based clustering in the proposed method. On the other hand, the performance of the proposed method under various channel conditions might also be investigated. As for future work, we could attempt to improve our method by exploring signal processing techniques that can effectively analyze wideband signals by dividing them into sub-bands. All these could be considered to be follow-up research.

## 7. Conclusions

This study proposes an STFT-based method to detect and estimate the parameters of multiple narrowband FH signals. It mainly involves four stages: (a) exploiting a binarization process based on threshold calculation to detect FH signals; (b) using a proposed algorithm to estimate the signal parameters; (c) adopting a combining algorithm that uses protection bandwidth to separate the center frequencies of the detected signals, (d) clustering and detecting based on the bandwidth parameter and the time overlap. Simulations were conducted to evaluate the efficiency of the proposed method at different SNR levels (−8 dB, 6 dB, and 24 dB). In the simulations, two FH signals with 4-FSK and 8-FSK modulation schemes generated by the GNU Radio library along with NumPy were used. The performance of the proposed method on the estimated signal parameters, such as center frequency, bandwidth, dwell time, guard time, and hopping rate, was assessed in terms of the absolute error metric. The results achieved from the simulations suggest that the proposed method could be a faster and more accurate alternative to mainstream STFT time–frequency analysis for the parameter estimation and detection of multiple narrowband FH signals.

## Figures and Tables

**Table 1:** Summary of relevant works.

Ref.	FH Parameters	Methods	Test Band	Number of FH Signals	FH Signal Properties
[4]	Center frequency Hopping time Hopping period DOA	STFD Joint diagonalization	-	3	Same hopping period and dwell time of an FH signal No overlap in frequency domain
[20]	Hop bandwidth Waveform Peak energy Dwell time	Adaptive threshold Time continuity detection Improved K-means clustering Chronological sorting	2.4 GHz	1–2	Same hopping period and dwell time of an FH signal
[24]	Center frequency Hop bandwidth Dwell time Modulation	Two-Stage Detection and Classification with GoF and DRNN Combined detection and classification with YOLO-lite	2.4 GHz	11	Overlap in frequency domain
[32]	Center frequency Hopping period Guard time	STFT and SPWVD	-	2	Same dwell time and different guard time of an FH signal No overlap in frequency domain
[33]	Center Frequency Hopping time	Segmentation CSML Averaging and zero-setting	-	2	Same hopping period and dwell time of an FH signal No overlap in frequency domain
[34]	Center frequency Hopping time Hopping period Hop bandwidth	DNN K-means clustering	-	1–2	Same hopping period and dwell time of an FH signal No overlap in frequency domain
[35]	Center frequency Hopping time Power	STFT Neural Network	-	4	Same hopping period and dwell time of an FH signal Different hopping time No overlap in frequency domain
[36]	Center frequency Hop bandwidth Dwell Time Hopping period	STFT FHExt extractor K-means clustering Deep learning	1.9–2.1 GHz	2,3,4	Same hopping period and dwell time of an FH signal Partial overlap in frequency domain
[37]	Center frequency Dwell time Hopping rate Hopping time	SPWVD Few-shot learning	-	1–6	Same hopping period and dwell time of an FH signal No overlap in frequency domain
[38]	Center frequency Hop bandwidth Hopping time Hopping period 2-D-DOA	STFD YOLO Beamspace transformation	-	5	Same hopping period and dwell time of an FH signal No overlap in frequency domain

**Table 2:** List of signal parameters.

FH ID	Modulation	$\tau_{dwell}$ (µs)	$\tau_{guard}$ (µs)	HR (hops/s)	$f_{center}$ (MHz)	BW (kHz)
FH-A	4-FSK	10,777.7	334.4	90	0.7	40
					10.7	
					20.7	
FH-B	8-FSK	7766.4	567	120	−15.5	80
					−5	
					5	
					15.5	

**Table 3:** Average absolute errors of the estimated parameters of the detected FH signals by varying K factors (SNR = 6 dB; Rician channel; K = 0.7, 1, 2.8, 10; $f_{D}$ = 100 Hz).

FH ID	K	$∆f_{center}$ (Hz)	∆BW (kHz)	$∆\tau_{dwell}$ (µs)	$∆\tau_{guard}$ (µs)	∆HR
FH-A	0.7	870	10	~29.43	8725	0.25
	1	~886.3	10	~29.43	8725	0.25
	2.8	872	10	~29.43	8725	0.25
	10	~887.6	10	~31.92	8725	0.275
FH-B	0.7	~1767.4	10	33.35	~33.64	0
	1	1788	10	33.35	~33.64	0
	2.8	~1747.8	10	33.35	~33.64	0
	10	1755	10	33.35	~33.64	0

## Data Availability

The data presented in this study are available on request from the corresponding author.
